# From Mum to Bum: An Observational Study Protocol to Follow Digestion of Human Milk Oligosaccharides and Glycoproteins from Mother to Preterm Infant

**DOI:** 10.3390/nu13103430

**Published:** 2021-09-28

**Authors:** Jannie G. E. Henderickx, Esther J. d’Haens, Marieke A. C. Hemels, Mariëtte E. Schoorlemmer, Astrid Giezen, Richard A. van Lingen, Jan Knol, Clara Belzer

**Affiliations:** 1 Laboratory of Microbiology, Wageningen University and Research, Stippeneng 4, 6708 WE Wageningen, The Netherlands; jannie.henderickx@wur.nl (J.G.E.H.); jan.knol@danone.com (J.K.); 2 Department of Neonatology, Isala Women and Children’s Hospital, Dokter van Heesweg 2, 8025 AB Zwolle, The Netherlands; e.j.haens@isala.nl (E.J.d.H.); m.a.c.hemels@isala.nl (M.A.C.H.); m.e.schoorlemmer@isala.nl (M.E.S.); a.giezen@isala.nl (A.G.); r.a.van.lingen@isala.nl (R.A.v.L.); 3 Danone Nutricia Research, Uppsalalaan 12, 3584 CT Utrecht, The Netherlands

**Keywords:** preterm, infant, microbiota, microbiome, nutrition, human milk, gastric aspirate, feces

## Abstract

The nutritional requirements of preterm infants are challenging to meet in neonatal care, yet crucial for their growth, development and health. Aberrant maturation of the gastrointestinal tract and the microbiota could affect the digestion of human milk and its nutritional value considerably. Therefore, the main objective of the proposed research is to investigate how the intestinal microbiota of preterm and full-term infants differ in their ability to extract energy and nutrients from oligosaccharides and glycoproteins in human milk. This pilot study will be an observational, single-center study performed at the Neonatal Intensive Care Unit at Isala Women and Children’s Hospital (Zwolle, The Netherlands). A cohort of thirty mother–infant pairs (preterm ≤30 weeks of gestation, *n* = 15; full-term 37–42 weeks of gestation, *n* = 15) will be followed during the first six postnatal weeks with follow-up at three- and six-months postnatal age. We will collect human milk of all mothers, gastric aspirates of preterm infants and fecal samples of all infants. A combination of 16S rRNA amplicon sequencing, proteomics, peptidomics, carbohydrate analysis and calorimetric measurements will be performed. The role of the microbiota in infant growth and development is often overlooked yet offers opportunities to advance neonatal care. The ‘From Mum to Bum’ study is the first study in which the effect of a preterm gut microbiota composition on its metabolic capacity and subsequent infant growth and development is investigated. By collecting human milk of all mothers, gastric aspirates of preterm infants and fecal samples of all infants at each timepoint, we can follow digestion of human milk from the breast of the mother throughout the gastrointestinal tract of the infant, or ‘From Mum to Bum’.

## 1. Introduction

Human milk is strongly recommended in infant feeding [1,2]. Besides its nutrient composition, human milk educates the neonatal immune system by promoting selective tolerance towards dietary and microbial components [3,4,5,6]. Human milk digestion starts with maternal enzymes in the breast that are subsequently accompanied by infantile enzymes in the mouth and stomach upon ingestion [7,8]. Further down the gastrointestinal tract, the microbiota in the colon fulfills an essential role in extracting nutrients from a considerable amount of food components that are otherwise indigestible, such as oligosaccharides and glycoproteins in human milk [9,10]. The process of human milk digestion is pivotal for development of the gastrointestinal tract, microbiota and immune system [1,11,12,13].

Digestion and absorption of human milk is impaired in preterm infants, having considerable consequences on their growth and development [13,14]. Besides physiological immaturity of the gastrointestinal tract, aberrant microbiota development impedes human milk digestion in preterm infants [13,14]. Preterm infants typically have a decreased microbial diversity compared to full-term infants, which has been shown to play a role in achieving weight gain [15,16,17]. Moreover, a differential microbiota composition may affect the abundance of the microbial gene pool encoding for proteins involved in metabolism of macronutrients, which subsequently would alter the metabolic activity and energy harvest [10,13,18,19,20]. Microbial digestive proteins have already been shown to vary with gestational and postnatal age in preterm infants [21,22]. Most convincing, however, are studies in preterm infants showing associations between the gut microbiota, growth and development in early life [20,23]. For example, various microbiota phases in preterm infants—each characterized by distinct metabolic functions—were significantly associated with preterm infant growth [20]. More specifically, levels of *Bacteroides*, *Enterobacteriaceae* and *Streptococcus* at early age could be associated with weight gain of preterm infants at one month of age [23]. 

With advances in neonatal care, the survival rates of preterm infants born at younger gestational ages have increased [24]. This imposes new clinical challenges such as meeting the unique nutritional requirements of preterm infants [8,25]. In fact, more than half of hospitalized preterm infants are being discharged with ongoing severe postnatal growth failure [20,26]. Growth impairment in the neonatal period is common and increases susceptibility to infections and impaired cognitive development [27,28]. The role of the microbiota in this process is often overlooked yet offers opportunities to advance neonatal care. Therefore, the metabolic capacity of the preterm gut microbiota and its subsequent role in infant growth and development should be investigated. 

### 1.1. The ‘From Mum to Bum’ Study

The new ‘From Mum to Bum’ pilot study is well-suited to investigate this and broadens our previous clinical set-up of the EIBER study. In the EIBER study, gastric aspirates and feces were collected from preterm and full-term infants with the main objective being to investigate the colonization and development of the gut microbiota [14,21,29,30]. The EIBER study has enabled us to study maturation of the gastrointestinal tract and the microbiota in the early life of preterm infants, as well as the relationship between microbiota composition and antibiotic treatment [14,21,29,30]. More specifically, gastrointestinal and beneficial microbial proteins involved in gastrointestinal maturity were associated with gestational and postnatal age [14,21]. In the new single-center, observational study at the Neonatal Intensive Care Unit (NICU) at Isala Women and Children’s Hospital (Zwolle, The Netherlands), we aim to achieve a targeted approach to compare the microbiota’s functionality of preterm and full-term infants with regard to the digestion of human milk components. To this end, a group of mother–infant pairs will participate during the first six postnatal weeks with follow-up at three- and six-months postnatal age. The group will consist of fifteen mothers delivering vaginally and full-term (37–42 weeks of gestation) and fifteen mothers delivering vaginally and preterm (≤30 weeks of gestation). By collecting human milk of all mothers, gastric aspirates of preterm infants and fecal samples of all infants at each timepoint, we can follow the digestion of human milk from the breast of the mother throughout the gastrointestinal tract of the infant, or ‘From Mum to Bum’. Previously, a similar set-up was used, in which human milk and corresponding infant feces were used to show that human milk oligosaccharides (HMOs) are important for shaping the gut microbiota of infants [31,32,33]. In the current study, gastric aspirates of preterm infants are included in sample collection, which will provide additional information on human milk digestion from a host perspective. Moreover, our study aims to integrate 16S rRNA amplicon sequencing, proteomics, peptidomics and carbohydrate analysis. With the integration of these methods, we can assess how the intestinal microbiota of preterm and full-term infants differ in their ability to extract energy and nutrients from oligosaccharides and glycoproteins in human milk. In fact, the combination of genomics and proteomics has been key in understanding that the bacterial digestive proteins of preterm infants vary with gestational age [21]. Moreover, the collection of multiple types of samples at each timepoint provides us with longitudinal data that allow us to follow microbial composition and host/microbial protein development during the first six postnatal months. Moreover, we will include calorimetric measurements to assess intestinal functionality.

### 1.2. Aim and Hypothesis

The main objective of the proposed research is to investigate how the intestinal microbiota of preterm and full-term infants differ in their ability to extract energy and nutrients from human milk. We expect that differences in gut microbiota of preterm infants will mainly be emphasized with regard to the digestion of HMOs and glycoproteins from human milk, since *Bifidobacterium* spp. are equipped with genes encoding for enzymes that digest these components and are lower in abundance in preterm infants [13,14,34].

Other aims are to: (1) identify the composition of microbiota in early life and its development over time; (2) assess the bifidogenic effect of human milk; (3) establish if there is a relationship between preterm microbiota composition, weight gain and growth in early life; and (4) explore the relationship between preterm microbiota composition and registered clinical variables.

## 2. Materials and Methods

### 2.1. Study Design and Setting

The ‘From Mum to Bum’ study is an observational, single-center pilot study that will include a cohort mother–infant pairs followed from birth until 6 months postpartum. The mother–infant pairs will comprise of mothers delivering preterm and full-term. The cohort will be recruited at the obstetrics department and NICU of Isala Women and Children’s Hospital (Zwolle, The Netherlands) as well as at several midwifery practices. Isala Women and Children’s Hospital is one out of nine hospitals with a level III NICU in the Netherlands. 

### 2.2. Sample Size Calculation

No published data are available to contribute to the estimation of the desired sample size. Therefore, a non-probabilistic, convenience sampling method will be applied over a period of two years. Based on the hospital’s statistics, it is expected that fifteen preterm mother–infant pairs, who fulfill the inclusion criteria and not the exclusion criteria, could be recruited within two years. The full-term mother–infant pairs group will be of equal size.

### 2.3. Recruitment Criteria

Subjects are eligible if they fulfill all the inclusion criteria, but not the exclusion criteria. Screening takes place when an infant is (to be) admitted to the NICU because of (suspected) preterm birth. Full-term subjects are recruited by midwives on a voluntary basis during pregnancy. Potential subjects are screened with respect to the inclusion and exclusion criteria. Written informed consent is obtained before inclusion in the study.

#### 2.3.1. Inclusion Criteria for Preterm Mother–Infant Pairs

The inclusion criteria for preterm mother–infant pairs are: (1) mothers who deliver ≤30 weeks of gestation and of whom the infants are admitted to the NICU at Isala Women and Children’s Hospital (Zwolle, The Netherlands); (2) the infant is born vaginally; (3) the infant has a nasogastric tube; and (4) there is an intention to breastfeed. 

#### 2.3.2. Inclusion Criteria for Full-Term Mother–Infant Pairs

The inclusion criteria for full-term mother–infant pairs are: (1) mothers who deliver between 37 and 42 weeks of gestation, of whom infants are born either in a hospital after an uncomplicated pregnancy or at home; (2) the infant is born vaginally; (3) there is an intention to breastfeed; and (4) both mother and infant are healthy, which is defined as not receiving any medication except vitamins. 

#### 2.3.3. Exclusion Criteria for (Pre)term Mother–Infant Pairs

Mother–infant pairs will be excluded if they do not meet the inclusion criteria. Other exclusion criteria include: (1) major congenital malformations (of the gastrointestinal tract) of the infant; (2) high probability of death within six weeks postpartum; (3) expected discharge from the NICU or transfer to another hospital during the first postnatal week; and (4) there is no intention to breastfeed and/or the infant does not receive any human milk after the first week postpartum. 

### 2.4. Sampling Procedures

#### 2.4.1. Data Collection Timeline

**Figure 1:**
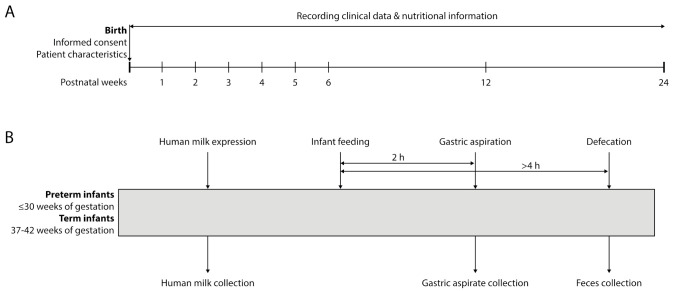
Sampling and data collection scheme. Scheme of (**A**) sampling points over the first six months and (**B**) one sampling point. While human milk and feces will be collected in full-term and preterm infants, gastric aspirates will only be collected in preterm infants during hospital stay. Clinical data will be monitored at every sampling point throughout the duration of the study.

The study will have a duration of six weeks and a follow-up at three- and six months postpartum. Samples will be collected weekly on the last day of the week during the first six weeks. Follow-up will occur on the last day of week 12 and week 24 (Figure 1A). Sample collection comprises: (1) human milk; (2) gastric aspirate (only in preterm infants); and (3) fecal sample of the infant (Figure 1B). In case of discharge from the hospital, human milk and fecal samples will be collected at home and frozen at −20 °C. Home collections will be transported by courier to Isala Women and Children’s hospital. 

#### 2.4.2. Human Milk

Human milk samples will be collected if the infant is exclusively fed with human milk or mixed fed. Before feeding the infant, 4 mL of expressed human milk will be collected by manual or mechanical expression. The sample will be stored at −20 °C until transfer to −80 °C for later analysis. Breastfeeding the infant will always be prioritized, and mothers will be encouraged to breastfeed their infants at all times as soon as the infant is able to drink from the breast; otherwise, gavage feeding of expressed human milk will take place. The amount of mother’s human milk will be registered in the Case Report Form (CRF). In case of insufficient human milk expression, infants will receive additional infant formula to complete the amount. If the mother cannot express human milk at all after the first week postpartum, mother-infant pairs will be excluded. No donor milk bank will be available at the NICU during the study period. For infants below 1800 g, human milk will be supplemented with human milk fortifier and vitamins according to the NICU protocol. In those cases, we will continue sample collection according to the protocol. 

#### 2.4.3. Gastric Aspirate

Preterm infants (≤30 weeks of gestation) admitted to the NICU will receive a nasogastric tube for gastrointestinal feeding as per usual. Generally, the contents of the stomach will be aspirated two hours after feeding to empty the stomach and prepare it for next feedings. From this gastric aspirate, 1 mL will be collected and frozen at −80 °C for later analysis. If no stomach content is available, this will be reported and other samples will be collected according to the protocol.

#### 2.4.4. Feces

Fecal samples will be collected from the first stool passed at least four hours after feeding. With a scoop attached to the sampling bottle, at least one scoop of feces will be collected. These samples will be stored at −20 °C and transferred to −80 °C for later analysis.

#### 2.4.5. Clinical Data Collection

After birth, clinical data of preterm and full-term infants will be registered and will comprise the gestational age, date of birth, mode of delivery, birth weight and parental data. During the hospital stay, the investigator will register the study parameters of the preterm infant weekly in a CRF at days of sampling and whenever applicable. The study parameters will include the date and time of measurement, body weight, length, head circumference, feeding regimen, feeding intolerance, morbidities, medication and respiratory support information. The feeding regimen data will include the volume of human milk, the volume of formula and data on nutritional support including parenteral and enteral feeding. In case of enteral feeding, human milk intake will be corrected for enteral feeding.

During home sampling at follow-up of the preterm infant group and for the full-term group in general, feeding information and anthropometric parameters will be registered in online questionnaires that will be sent at the planned time of home sampling. Feeding information will include the volume of human milk and formula given to the infant at each sampling point. 

### 2.5. Primary Outcome

The main objective of the proposed research is to investigate how the intestinal microbiota of preterm and full-term infants differ in their ability to extract energy and nutrients from human milk. As such, the primary outcome will be the combination of quantitative differences between preterm and full-term infants in (1) HMO-degrading bacteria; (2) bacterial HMO-degrading enzymes; (3) human- and bovine-derived proteins; and (4) intestinal absorption capacity. This will be assessed in human milk, gastric aspirates and fecal samples collected during the first six postnatal weeks and a follow-up at three and six months.

### 2.6. Secondary Outcomes

- Microbiota composition in early life and its development over time, assessed using 16S rRNA gene amplicon sequencing and quantitative PCR (qPCR).
- The effect of (corrected) human milk intake on the relative abundance of *Bifidobacterium* spp.
- The relationship between preterm microbiota composition and weight gain in early life assessed by means of anthropometrics (weight, length and head circumference) and 16S rRNA gene amplicon sequencing.
- The relationship between preterm microbiota composition and registered clinical variables.

### 2.7. Sample and Data Processing

#### 2.7.1. Total Carbohydrates and Human Milk Oligosaccharides

Chemical analyses will be used to assess the compounds present in human milk, gastric aspirates and fecal samples. Specifically, the identity and quantity of carbohydrates present in human milk, gastric aspirates and feces will be analyzed by gel permeation chromatography (GPC) as described by Chia et al. [35].

HMOs will be measured by Liquid Chromatography Electrospray Ionization Tandem Mass Spectrometry (LC-ESI-MS^2^) as described by Mank et al. [36]. Pre-treatment of samples for this method will depend on the type. Human milk and gastric aspirates will be processed according to Mank et al. [36]. Briefly, samples will be thawed on ice and vortexed. Quantities of 15 µL of internal standard α-L-arabinopentaose (0.05 mM) will be added to 135 µL human milk or gastric aspirate. The solution of the sample and the internal standard will be further diluted 1:11 (*v*/*v*) through the addition of 150 µL Pierce™ Water, LC-MS Grade (ThermoFisher Scientific, Waltham, United States, Cat. No. 51140). Subsequently, 450 µL of diluted sample will be transferred to a 500-μL Amicon Ultra centrifugal filter with 3-kDa cutoff and ultrafiltration (UF) will be performed at 14,000× *g* for 1 h. Subsequently, 300 µL of UF permeate will be transferred to a LC-MS screw top vial for LC-MS analysis. The protocol will be slightly adapted for fecal samples, as suggested by Mank et al., and would include ‘additional microfiltration steps or SPE (…) in addition or as an alternative to 3-kDA ultrafiltration.’ [36]. Acquired data will be processed as described by Mank et al. [36]. Processed data will be used for data analysis.

#### 2.7.2. Metaproteomic and Peptidomic Analysis

The metaproteome of human milk, gastric aspirate and feces will be characterized using LC-MS/MS according to the methods outlined by Zwittink et al. [21]. For peptidomics, the samples will be prepared and analyzed according to Dallas et al. [37].

Metaproteomics and peptidomics data will be processed with MaxQuant [38] and further processed in Perseus as described previously [21]. Label-free quantification (LFQ) intensities will be log_10_-transformed. Intensity-Based Absolute Quantification (iBAQ) intensities will be used to measure the relative abundance of proteins. Functional profiles of proteins will be generated by assigning protein IDs to KEGG Orthology (KO) identifiers using the KEGG Brite database. Processed data will be used for data analysis.

#### 2.7.3. Microbiota Analysis

16S rRNA gene amplicon sequencing be used to assess the microbiota composition and relative abundance in human milk, gastric aspirates and feces. For microbiota analysis, bacterial DNA will be isolated from feces. Quantities of 0.13 g of feces will be weighed into a 2.0 mL screw cap tube filled with 0.25 g of 0.1 mm zirconia beads and three 2.5 mm glass beads. Negative controls will be included and consist of FastPrep tubes with beads. Furthermore, 300 µL of Stool Transport and Recovery Buffer (S.T.A.R. buffer, Roche Diagnostics, Almere, The Netherlands, Cat. No. 03335208001) will be added and bead-beaten three times at 5.5 ms for 60 s with 15 s pause (FastPrep-24™ 5G bead beating grinder and lysis system, MP Biomedicals, Irvine, United States). Subsequently, samples will be incubated for 15 min at 95 °C at 100 rpm, after which they will be centrifuged (4 °C, 5 min 14,860 rpm) and the supernatant will be stored at 4 °C. The process will then be repeated with 200 µL S.T.A.R. buffer. In case the first step does not yield supernatant, 300 µL S.T.A.R. buffer will be added. A total of 250 µL of recovered supernatant will be used for DNA extraction with Maxwell^®^ 16 Tissue LEV Total RNA Purification Kit (Promega, Wisconsin, United States Cat. No. AS 1220).

Isolated DNA will be PCR-amplified with barcoded V4 primers (515F: GTGYCAGCMGCCGCGGTAA [39]; 806R: GGACTACNVGGGTWTCTAAT [40]). Next, PCR products will be purified with the CleanPCR kit (CleanNA, Waddinxveen, The Netherlands, Cat. No. CPCR-0050) according to the manufacturer’s protocol. DNA will be quantified with the Qubit™ dsDNA BR Assay Kit (ThermoFisher Scientific, Waltham, United States, Cat. No. Q32850) on DeNovix DS-11 FX (DeNovix, Wilmington, United States) and pooled into libraries at an equimolar concentration of 200 ng. The pooled products will be purified with the CleanPCR kit according to the manufacturer’s protocol and sequenced with the Illumina HiSeq platform.

Sequencing data will be annotated with the SILVA database using our in-house NG-Tax pipeline with default settings. In short, NG-Tax will perform read filtering, Amplicon Sequence Variant (ASV)-picking and taxonomic assignment. The processed data will be used for data analysis.

A subset of bacterial families and genera of interest will additionally be quantified using a SYBR-based real-time qPCR. The subset of microorganisms will be selected based on reported core microbiota in preterm infants and on their involvement in the degradation of components in human milk [12,21,34,41]. The subset of bacterial families and genera will include the *Enterobacteriaceae* family and the genera *Bacteroides*, *Bifidobacterium*, *Clostridium*, *Enterococcus* and *Lactobacillus*. Primer sequences will be used to target the family- or genus-specific regions of the bacterial 16S rRNA gene (Table 1). Instead of genus-specific primers, phylogenetic cluster XIVa will be selected as target for the *Clostridium* genus as the 16S rRNA gene shares great homology between strains [42]. The selected cluster is among the most abundant *Clostridium* phylogenetic clusters that have been identified in the human gastrointestinal tract [43].

#### 2.7.4. Calorimetry

The energy contained within human milk and feces will be measured using bomb calorimetry, as described earlier [49,50,51,52,53]. Intestinal absorption capacity will by defined by the energy difference between nutritional intake and fecal losses, which is a widely accepted method and semi-quantitative marker of intestinal function in clinical practice [49]. Human milk will be used to measure nutritional intake and feces will be used to measure the energy excreted in feces. Analyses will be performed according to Hosoi et al. for human milk [51] and Wierdsma et al. for feces [49]. 

### 2.8. Data Availability

Once available, the mass spectrometry data will be deposited to the ProteomeXchange Consortium via the PRIDE partner repository. Sequencing data will be made available via the European Nucleotide Archive.

### 2.9. Ethics Approval and Consent to Participate

The protocol for the ‘From Mum to Bum’ study was approved by the board of the Medical Ethics Committee (METC) of Isala Zwolle in May 2019 as a study not falling under the scope of the Medical Research Involving Human Subjects Act (WMO). The study was registered under the number 190503 with the Research Manager of METC Isala Zwolle and began recruiting in August 2020. This study will be conducted according to the principles of the Declaration of Helsinki (64th WMA General Assembly, Fortaleza, Brazil 2013), the Personal Data Protection Act (UAVG), the ‘Gedragscode Gezondheidsonderzoek,’ and the ‘Code Goed Gedrag’.

### 2.10. Data Management

The privacy of the participants will be guaranteed at all times. The data of participating infants will be pseudonymized with personal codes. Samples and registered data will be collected in the CRF using this code. The document linking codes to participants’ data will only be accessible for the researchers of this study. The investigator is responsible for designing and updating the CRF and other data collection forms. All documents pertaining to the conduct of the study must be kept by the investigator for a period of 15 years.

## 3. Results

### 3.1. Data Analysis and Assessments

Subjects with missing values will be excluded prior to data analysis. Data will be analyzed using the statistical program R and RStudio software [54], as well as dedicated in-house R-scripts and available packages.

#### 3.1.1. Carbohydrates and Oligosaccharides

Preterm and full-term infants will be compared with regard to the quantity of total carbohydrate and the quantity of HMOs in their respective postnatal week, as well as within one age group between sample types. In addition, temporal dynamic plots will be used to assess the quantity of total carbohydrates and HMOs over the first six postnatal weeks.

#### 3.1.2. Microbiota Data

16S rRNA gene amplicon sequencing data over time will be analyzed in terms of composition, diversity and richness. Descriptive statistics such as summaries and graphics will be used to describe the basic features of the colonization and development of the gut microbiota of the subjects. The diversity and richness of the microbial groupsg within individuals will be analyzed at various phylogenetic levels using the paired Wilcoxon test. Differences in microbial composition, diversity and richness between time points will be assessed using a repeated measure Analysis of Variance (ANOVA) if the data are normally distributed or a Kruskal–Wallis test when the data are skewed. qPCR data will be used to assess the microbial load in each sample.

#### 3.1.3. Metaproteomics and Peptidomics

Proteins and peptides will be compared between the preterm and full-term groups in their respective postnatal week, as well as within one age group between sample types, using Perseus’ volcano plots [55]. The quantities of proteins and peptides of interest will be further analyzed with temporal dynamic plots over the first six postnatal weeks.

#### 3.1.4. Calorimetry

Measured energy (kcal/100 g) and intestinal absorption capacity (as a percentage of nutritional intake) will be compared between preterm and full-term groups in their respective postnatal week as well as within one age group over the first six postnatal weeks.

#### 3.1.5. Relationships between Data

Metaproteomic and 16S rRNA gene amplicon sequencing will be further analyzed in relation to clinical variables. Considering all measured variables, principal component analysis (PCA) will be used to assess the captured variation between groups. Moreover, this technique allows us to examine potential clusters and outliers. Next, redundancy analysis (RDA) will be used to estimate the relationship between microbiota composition and other quantitative and qualitative variables including 16S rRNA sequencing data, metaproteomics data and clinical variables. Forward and reverse automatic stepwise model selection for constrained ordination will be performed to build a model with variables that significantly explain variation in the data. Additionally, correlation network analyses will be performed between the relative abundance of gut bacteria, human/bacterial proteins and the clinical variables. 

## 4. Discussion

The ‘From Mum to Bum’ study is a new clinical pilot study investigating how the intestinal microbiota of preterm and full-term infants differ in their ability to extract energy and nutrients from oligosaccharides and glycoproteins in human milk. It capitalizes upon the set-up of our previous clinical trial (EIBER) and broadens it by including mother’s human milk in the sample collection. The inclusion of human milk is crucial to advance the understanding of the digestion of human milk, from the breast of the mother, throughout the gastrointestinal tract of the infant. 

The microbiota plays a central role in this study, as it is often overlooked in nutritional neonatal care [13]. The ‘From Mum to Bum’ study is the first study in which the metabolic capacity of the preterm gut microbiota and subsequent infant growth and development is investigated. We aim to unravel microbial degradation of oligosaccharides and glycoproteins present in human milk along the gastrointestinal tract. The proposed research is innovative in terms of the collection of samples obtained at multiple sites along the gastrointestinal tract. Human milk, gastric aspirates and feces have previously been studied in relation to microbial human milk digestion, but our study is the first to combine all three types of samples. Previously, intact HMOs and glycan digestion products have been quantified and characterized in human milk and/or feces [56,57,58,59,60,61,62]. Others have characterized and compared peptides in human milk and gastric aspirates [7,37]. However, these studies have not used a combination of human milk, gastric aspirates and feces in preterm infants. Another innovative aspect is the investigation of the microbial metabolic capacity in relation to anthropometric data, which only few studies have focused on [20,23]. Moreover, we will be able to follow this process during the first six postnatal weeks. 

We acknowledge a few limitations of this study. First, the single-center set-up of the study may compromise the feasibility of recruiting solely preterm infants that are born vaginally. The mode of delivery has been identified to strongly influence microbiota composition in (preterm) infants [63,64]. Selecting infants with the same mode of delivery, therefore, eliminates differences in microbiota composition due to confounding factors. Yet, more frequently than full-term infants, preterm infants are born via caesarean section and this group may, thus, not be represented by the cohort within this study [13]. Additionally, preterm infants are a heterogeneous group with many clinical variables acting as confounding factors. Selecting for mode of delivery does not exclude the effects of other confounding factors. Second, the sample size is based on a non-probabilistic, convenience sampling method but it remains unknown whether this sample size is large enough to capture heterogeneity in microbiota composition amongst preterm infants. Third, the collection of data from full-term infants relies heavily upon the compliance of participating parents. Questionnaires need to be filled out weekly by the parents in order to inquire about infant feeding practices. Additionally, human milk and feces need to be collected weekly and stored in the correct way to allow for microbiota analysis. Storage conditions, including temperature, have been shown to influence human milk peptidome and fecal microbiota composition [65,66,67,68]. Lastly, the absorption of proteins from human milk in the small intestine cannot be measured directly, although it may influence metabolic activity of the microbiota in the colon. 

With increasing survival rates at lower gestational ages, the feeding of preterm infants with unique nutritional requirements has become a new clinical challenge [8,24,25]. We expect that insights from this study can be used to tailor nutritional care to preterm infants in such a way that optimal growth and development can be enforced, which is beneficial for short- and long-term health.

## 5. Conclusions

In summary, the ‘From Mum to Bum’ study aims to investigate how the intestinal microbiota of preterm and full-term infants differ in their ability to extract energy and nutrients from human milk. By collecting human milk of the mother and gastric aspirates and feces of the infant, we can determine human milk composition, gastric digestion by the infant and fermentation by the intestinal microbiota of the infant. This may aid in the optimization of currently used feeding regimens, and could thus contribute to reductions in morbidity, mortality and health care costs. Additionally, the innovative methods from this study could be used to study the digestion of bovine milk components, and thereby contribute to developments in preterm infant formulas tailored to fit the needs of this group of infants.

## Figures and Tables

**Table 1:** Primer sequences targeting genera within the preterm core microbiota. Overview of primer sequences to target genus-specific regions of the bacterial 16S rRNA gene for genera within the preterm core microbiota. The subset of selected microorganisms is based on reported core microbiota in preterm infants and on their involvement in the degradation of components in human milk. References of primer sequences and associated methodology are included.

Target		Name	Sequence (5′–3′)	Amplicon Length (bp)	Tm	Reference
16S	Forward	BACT_1369F	CGG TGA ATA CGT TCY CGG	142	56	Suzuki et al. [44]
	Reverse	PROK_1492R	GGW TAC CTT GTT ACG ACT T			
*Bacteroides-Prevotella-Porphyromonas*	Forward	-	GGT GTC GGC TTA AGT GC CAT	140	68	Jian et al. [45]
	Reverse	-	CGG AYG TAA GGG CCG TGC			
*Bifidobacterium* spp.	Forward	-	TCG CGT CYG GTG TGA AAG	243	58	Jian et al. [45]
	Reverse	-	CCA CAT CCA GCR TCC AC			
*Clostridium* cluster XIVa	Forward	-	CGG TAC CTG ACT AAG AAG C	429	55	Jian et al. [45]
	Reverse	-	AGT TTY ATT CTT GCG AAC G			
*Enterobacteriaceae* spp.	Forward	En-lsu-3F	TGC CGT AAC TTC GGG AGA AGG CA	428	60	Matsuda et al. [46]
	Reverse	En-lsu-3′R	TCA AGG ACC AGT GTT CAG TGT C			
*Enterococcus* spp.	Forward	g-Encoc-F	ATC AGA GGG GGA TAA CAC TT	337	55	Matsuda et al. [47]
	Reverse	g-Encoc-R	ACT CTC ATC CTT GTT CTT CTC			
*Lactobacillus* spp.	Forward	F_alllact_IS	TGG ATG CCT TGG CAC TAG GA	92	58	Haarman et al. [48]
	Reverse	R_alllact_IS	AAA TCT CCG GAT CAA AGC TTA CTT AT			
	Probe	P_alllact_IS	TAT TAG TTC CGT CCT TCA TC		68	

## Data Availability

No new data were created or analyzed in this study. Data sharing is not applicable to this article.
